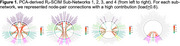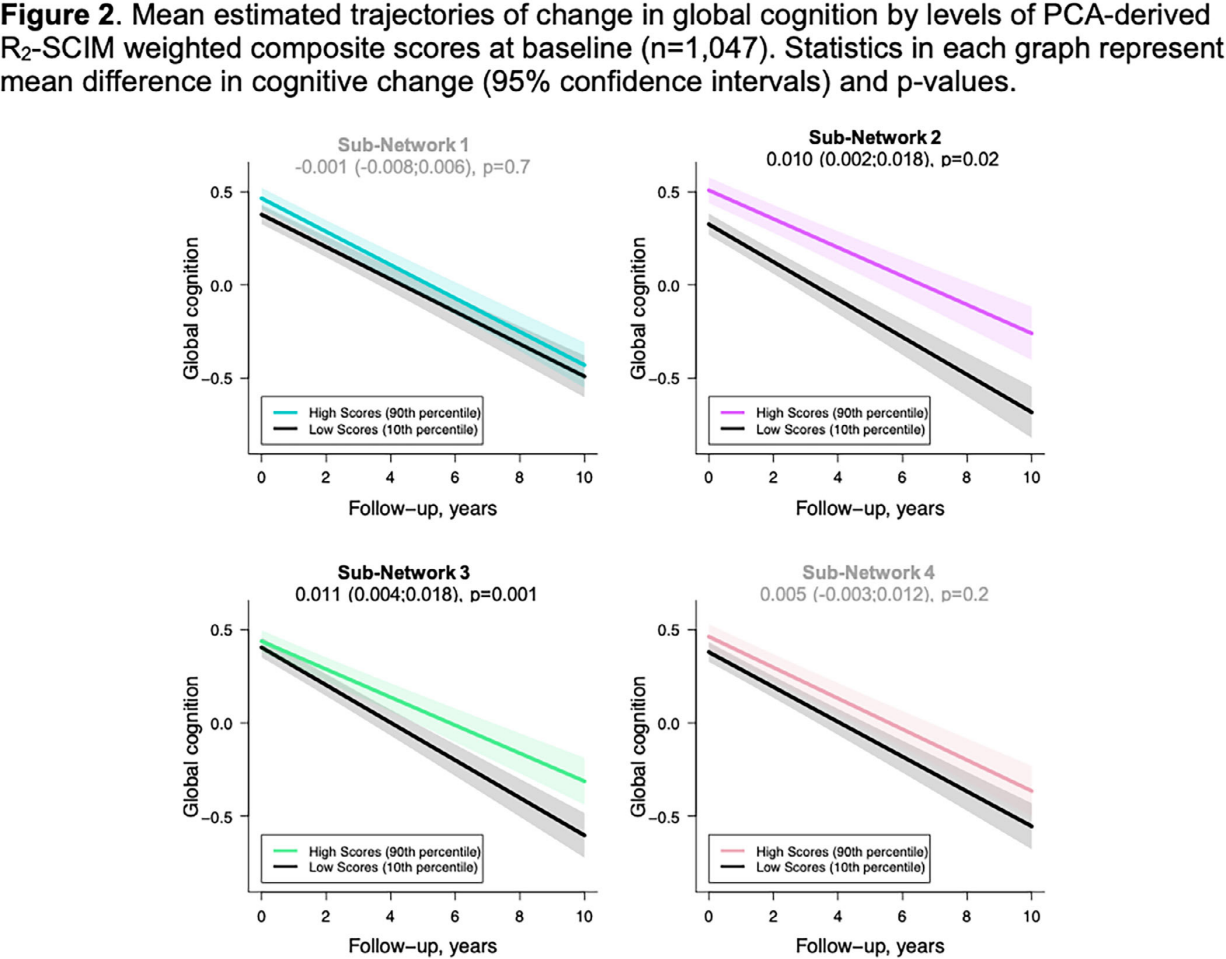# Derivation of a structural connectome integrity matrix based on the complex neural circuitry of the aging brain: A multi‐modal perspective applied to cognitive aging

**DOI:** 10.1002/alz70856_105182

**Published:** 2026-01-10

**Authors:** Melissa Lamar, Maude Wagner, Shengwei Zhang, Sue E. Leurgans, Victoria N Poole, Lisa L. Barnes, David A. A. Bennett, David X. Marquez, Julie A Schneider, Konstantinos Arfanakis

**Affiliations:** ^1^ Rush Alzheimer's Disease Center, Rush University Medical Center, Chicago, IL, USA; ^2^ Rush University Medical Center, Chicago, IL, USA; ^3^ Rush Alzheimer's Disease Center, Chicago, IL, USA; ^4^ Department of Kinesiology and Nutrition, University of Illinois Chicago, Chicago, IL, USA; ^5^ Department of Biomedical Engineering, Illinois Institute of Technology, Chicago, IL, USA

## Abstract

**Background:**

Structural connectome‐focused neuroimaging provides an important window into the aging brain; however, few studies address the complexities of mapping structural connectivity in late‐life. Of those that do, most either employ ‘lesion‐filling’ approaches to calculate connectivity metrics, or statistically adjust for lesions. These reasonable applications of connectome metrics to older adults with brain abnormalities and/or focal lesions may nonetheless obscure the impact of such lesions on (dis‐) connectivity and cognitive impairment.

**Methods:**

We applied alternative solutions to 3T neuroimaging data of 1,047 Rush Alzheimer's Disease Center cohort participants [age(years)≈78±7; 61% non‐Latino White]. We used an atlas‐based definition of the path of white matter connections via a structural connectivity‐based atlas, data‐driven network edge selection, and multi‐modal MRI metrics to reveal subtle distinctions in structural connectome integrity. Global cognition was assessed annually using 19 cognitive measures.

**Results:**

Data‐driven network edge selection resulted in 308 major edges contributing to the overall structural connectome integrity matrix (SCIM). Separate principal component analyses (PCAs) of MRI‐derived transverse relaxation rates (R_2_), fractional anisotropy, and quantitative susceptibility mapping revealed modality‐specific sub‐networks. For example, the R_2_‐SCIM PCA revealed four sub‐networks containing U‐fibers within lobes and connections between lobes (Figure 1): Sub‐Network 1 was characterized by R_2_ integrity within edges involving most frontal nodes (i.e., grey matter regions), all parietal nodes, and key subcortical structures including the basal ganglia; Sub‐Network 2 involved most albeit slightly different frontal nodes than Sub‐Network 1, nearly all temporal nodes, and key subcortical structures including limbic structures; Sub‐Network 3 was primarily characterized by R_2_ integrity of edges involving select parietal and temporal nodes and all occipital nodes; Sub‐Network 4 involved only the cerebellum, basal ganglia and limbic structures. A linear mixed‐effects regression of global cognition containing PCA‐derived weighted composite scores representing each R_2_‐SCIM sub‐network and relevant confounders demonstrated associations of higher R_2_ in Sub‐Networks 1, 2, and 4 with higher cognition at baseline (*p*‐values≤0.045) and associations of higher R_2_ in Sub‐Networks 2 and 3 with slower decline in cognition (*p*‐values≤0.02) (Figure 2).

**Conclusion:**

Our approach to understanding the aging connectome provides a comprehensive assessment of structural connectome integrity, its underlying sub‐networks, and their associations with cognition.